# CD8 T Cell Tolerance to a Tumor-Associated Self-Antigen Is Reversed by CD4 T Cells Engineered To Express the Same T Cell Receptor

**DOI:** 10.4049/jimmunol.1401703

**Published:** 2014-12-24

**Authors:** Sara Ghorashian, Pedro Veliça, Ignatius Chua, Anne-Marie McNicol, Ben Carpenter, Angelika Holler, Emma Nicholson, Maryam Ahmadi, Mathias Zech, Shao-An Xue, Wolfgang Uckert, Emma Morris, Ronjon Chakraverty, Hans J. Stauss

**Affiliations:** * Institute of Immunity and Transplantation, University College London, Royal Free Hospital, London NW3 2PF, United Kingdom;; † Transplantation Immunology Group, Department of Haematology, Division of Cancer Studies, University College London, London NW3 2PF, United Kingdom; and; ‡ Institute of Biology, Humboldt University Berlin and Max Delbrück Center for Molecular Medicine, 13125 Berlin, Germany

## Abstract

Ag receptors used for cancer immunotherapy are often directed against tumor-associated Ags also expressed in normal tissues. Targeting of such Ags can result in unwanted autoimmune attack of normal tissues or induction of tolerance in therapeutic T cells. We used a murine model to study the phenotype and function of T cells redirected against the murine double minute protein 2 (MDM2), a tumor-associated Ag that shows low expression in many normal tissues. Transfer of MDM2-TCR–engineered T cells into bone marrow chimeric mice revealed that Ag recognition in hematopoietic tissues maintained T cell function, whereas presentation of MDM2 in nonhematopoietic tissues caused reduced effector function. TCR-engineered CD8^+^ T cells underwent rapid turnover, downmodulated CD8 expression, and lost cytotoxic function. We found that MDM2-TCR–engineered CD4^+^ T cells provided help and restored cytotoxic function of CD8^+^ T cells bearing the same TCR. Although the introduction of the CD8 coreceptor enhanced the ability of CD4^+^ T cells to recognize MDM2 in vitro, the improved self-antigen recognition abolished their ability to provide helper function in vivo. The data indicate that the same class I–restricted TCR responsible for Ag recognition and tolerance induction in CD8^+^ T cells can, in the absence of the CD8 coreceptor, elicit CD4 T cell help and partially reverse tolerance. Thus MHC class I–restricted CD4^+^ T cells may enhance the efficacy of therapeutic TCR-engineered CD8^+^ T cells and can be readily generated with the same TCR.

## Introduction

Adoptive transfer of T cells genetically engineered to express TCRs for tumor-associated Ags (TAAs) is actively being explored as therapy for cancer (1–4). Candidate Ags are assessed by criteria such as their immunogenicity, expression levels within neoplastic compared with normal cells, and whether they have shared expression in patients with different tumor types (5). Targeting TAAs derived from proteins with a direct role in neoplastic transformation is attractive because this may prevent development of Ag-loss variants that escape T cell attack. Unfortunately, many of these proteins are also expressed in normal tissues. Targeting of such Ags for therapeutic purposes may cause detrimental autoimmune damage, or it may induce unresponsiveness of adoptively transferred T cells due to chronic Ag exposure. In this study we analyzed to what extent the expression of TAAs in normal tissues impairs T cell function in vivo, and whether it is possible to develop strategies to reverse this.

The murine double minute protein 2 (MDM2) oncogene is required for cellular transformation through its function in inactivating the p53 tumor suppressor protein (6, 7). Although overexpressed in many cancers, it is also found in normal tissues, albeit at lower levels (6–8). As a consequence, high-avidity MDM2-specific T cells are deleted from the repertoire in the thymus or become subject to peripheral tolerance mechanisms (9). To bypass self-tolerance, we previously used an allorestricted strategy to generate high-avidity allo-MHC–restricted CTLs specific for peptide epitopes of MDM2 in both human (9) and murine (10) T cell repertoires. A murine MDM2-derived peptide, pMDM100, that is naturally presented on H2-K^b^ MHC class I (MHC-I) molecules, is recognized by high-avidity allorestricted MDM2-specific CTL clones from H2^d^ BALB/c mice (10). We have previously demonstrated that whereas naturally presented K^b^/pMDM100 peptide in normal hematopoietic cells is insufficient to induce killing, endogenous presentation of K^b^/pMDM100 in several tumor lines readily triggers Ag-specific cytotoxicity (10, 11). However, although the CTL clones can induce potent antitumor effects in vivo, they become rapidly exhausted under conditions where Ag is also expressed in normal tissues (11). In a therapeutic setting, this loss of function may reduce antitumor efficacy.

Provision of CD4^+^ T cell help (Th) during primary or recall responses, or during chronic exposure to Ag, has been demonstrated to enhance CD8^+^ T cell–mediated immunity (12, 13). Th responses augment CTL functions directly through release of effector cytokines or indirectly through licensing of dendritic cells (DCs) (13). However, application of this approach in the clinic has been limited by the paucity of validated MHC class II (MHC-II)–restricted TAAs and/or the lack of expression of MHC-II in cancer cells (14). One potential means of overcoming these barriers is the redirection of CD4^+^ T cell specificity through gene transfer of MHC-I–restricted TCRs that recognize TAAs (15–17). CD4^+^ T cells engineered in this way can proliferate and release Th cytokines in response to MHC-I peptide ligand (15–17). We have shown previously that CD4^+^ T cells modified to express an influenza-specific MHC-I–restricted TCR can provide help in vivo to CD8^+^ T cells expressing the identical TCR (17). However, in this model Ag system, the CD8^+^ T cell population was not affected by exposure to naturally expressed TAA in normal tissues. We therefore sought to determine how chronic Ag exposure affects the functional profile of CD4^+^ and CD8^+^ T cells expressing the same TAA-specific TCR, and whether such CD4^+^ T cells can help to overcome Ag-related dysfunction in CD8^+^ T cell populations.

In this study, we find that transfer of a high-avidity, MDM2-specific TCR into CD8^+^ T cells leads to a rapid loss of their fitness as a consequence of Ag recognition upon self. T cell exhaustion in Ag-expressing hosts can be partially reversed by cotransfer of CD4^+^ T cells engineered to express the identical TCR, where the absence of the CD8 coreceptor enables preservation of helper function.

## Materials and Methods

### Mice and cell lines

C57BL/6 (B6), B6.PL (Thy1.1), B6.SJL (CD45.1), BALB/c, B6 × BALB/c F_1_ (CB6F1), B6 *Rag1^−/−^* (B6.129S7-*Rag1^tm1Mom^*/J), BALB/c Thy1.1 (By.PL(B6)-*Thy1^a^*/ScrJ), and *Rag2^−/−^* OT-II mice were purchased from Harlan Laboratories (Loughborough, U.K.), Charles River Laboratories (Wilmington, MA), or The Jackson Laboratory (Bar Harbor, ME) and maintained in the University College London animal facility. Experiments were performed in accordance with Home Office (Animals Scientific Procedures Act 1986) and local ethics committee guidance. RMA-S cells (H2^b^) are a T cell lymphoma cell line and are TAP-deficient (18). Ecotropic Phoenix packaging cells (PEco) used for retroviral particle production were a gift from Dr. G.P. Nolan (Stanford University, Stanford, CA).

### Peptides

The peptide pMDM100 (YAMIYRNL) binds to H2-K^b^ MHC-I (19) and the peptide pNP366 (ASNENDAM) of influenza virus A nucleoprotein (NP) binds to H2-D^b^ MHC-I (20). The peptide pOVA323 (ISQAVHAAHAEINEAGR) of chicken egg OVA binds to I-A^b^ and is recognized by the OT-II TCR. All peptides were generated by ProImmune (Oxford, U.K.).

### Retroviral constructs

*Tcra* and *Tcrb* genes were cloned as described previously from the influenza A NP–specific F5 CTL clone recognizing pNP366 (21) as well as from the pMDM100-specific CTL clones 3f3b (high avidity) and 6a5d (low avidity) (22). *Tcra* and *Tcrb* genes were codon optimized and two c-myc tag sequences inserted downstream of the *Tcra* leader sequence (23). Additional disulfide cysteine bonds were engineered between the TCRα and TCRβ constant regions and each gene was separated by a 2A sequence before cloning into the pMP71 retroviral vector, a gift from Dr. C. Baum (Medical School Hannover, Hannover, Germany). For experiments requiring cotransduction with CD8αβ, *CD8a* and *CD8b* genes separated by a 2A sequence were cloned into pMP71. Murine T cells were transduced as described previously (22).

### Adoptive transfer of transduced lymphocytes

Purified CD8^+^ and CD4^+^ T cells were transduced with each retroviral construct. Three days later, cells were analyzed by FACS for TCR expression and either injected as bulk populations or sorted on a BD FACSAria flow cytometer to generate pure CD8^+^ TCR-expressing (c-myc^+^ or Vβ11^+^) populations before transfer. Recipient mice were irradiated (5.5 Gy) 4 h before i.v. injection of 0.1–1 × 10^6^ TCR-transduced cells. Cell numbers were corrected for TCR expression in each experiment. Bone marrow–derived DCs were generated as described (24). In some experiments, 100 mg anti-CD8–depleting Ab (2.43, Bio X Cell, West Lebanon, NH) was administered i.p. at weekly intervals.

### Bone marrow transplantation

Hematopoietic chimeric animals were generated as described previously (25). Recipient mice were lethally irradiated (B6, 11 Gy; CB6F1, 10 Gy; BALB/c, 8 Gy) and reconstituted with 5 × 10^6^ recipient-type or 15 × 10^6^ allogeneic T cell–depleted bone marrow cells. In some experiments, BALB/c (K^b^-negative) T cells were transduced and adoptively transferred to recipient chimeras. For this, it was necessary to derive BALB/c origin T cells that would not mount alloresponses to K^b^. Thus, CB6F1 mice were lethally irradiated and reconstituted with 10 × 10^6^ T cell–depleted BALB/c bone marrow cells and 10 × 10^6^ unselected bone marrow cells from B6 *Rag1*^−/−^ mice. Chimeric animals were allowed to reconstitute stable hematopoiesis for a minimum of 12 wk before being used either as splenocyte donors or recipients for adoptive transfer. Where BALB/c origin T cells were derived from chimeric animals, K^b^-expressing splenocytes were depleted by magnetic bead separation after preincubating with anti–H2-K^b^ Ab (AF6-88.5, BD Pharmingen). Transduction of these splenocytes was then carried out as already described.

### Flow cytometry

The following mAbs and isotype controls were obtained from eBioscience (Hatfield, U.K.): anti-CD8b (H35-17.2), anti-CD4 (GK1.5), anti-Thy1.1 (HIS51), anti-CD45.1 (A20), anti-CD45.2 (104), and anti-Vβ11 (RR3-15). We obtained the following mAbs and isotype controls from BD Pharmingen: anti-CD44 (IM7), anti-CD62L (MEL-14), anti–programmed death 1 (PD-1; J43), and anti-CD69 (H1.2F3). A murine anti–c-myc (9E10, Santa Cruz Biotechnology) and a secondary rat anti-murine IgG Ab (X56, BD Pharmingen) were used to assess TCR α-chain expression. Samples were run on a BD LSRFortessa (BD Biosciences, Oxford, U.K.) and analysis was performed using FlowJo software (Tree Star).

Transduced T cell populations were identified based on a live lymphocyte gate, followed by expression of a congenic marker (where relevant), expression of a coreceptor or coreceptors (CD4 and/or CD8), and then by expression of the c-myc tag (for MDM2- or LoMDM2-TCR–transduced populations) or by expression of Vβ11 (F5-TCR–transduced populations). Where intensity of CD8 expression was investigated, this was excluded from the initial gating strategy, and transduced populations were identified from expression of the relevant congenic marker as well as the introduced TCR.

### Ag-specific cytokine production

ELISA was used to measure IFN-γ and IL-2 concentrations, as described previously (17).

### *^51^*Cr-release and in vivo cytotoxicity assays

Cytotoxic activity was determined using a 4-h ^51^Cr-release assay. In vivo cytotoxicity was performed as described previously (22, 26). In brief, B cells were isolated from syngeneic splenocytes, divided into equal aliquots, and loaded with either the pMDM100 or pNP366 peptides. The NP peptide–loaded B cells were stained with 0.5 μM and the MDM2 peptide–loaded B cells were loaded with 5 μM CFSE. The B cell aliquots were mixed at a 1:1 ratio. Total B cells (1 × 10^7^) were then injected i.v. into recipient mice after prior adoptive transfer of TCR-transduced T cells. The relative killing of these differentially labeled B cells was then determined by flow cytometry of splenocyte preparations after 16 h, and the specific lysis of B cell targets was calculated as described previously (22, 26).

### Histological analysis

Histological sections of skin, liver, lung, and colon from recipients of adoptively transferred T cells were stained with H&E and were assessed for evidence of immunopathology in a single blinded fashion. Images of representative sections were taken on an Olympus IX70 microscope fitted with a Canon EOS 1000D camera, using either a ×10 (skin, liver, lung) or ×20 (colon) objective lens.

### Statistical analysis

Statistical comparisons involved the Mann–Whitney two-tailed test. A *p* value <0.05 was considered significant.

## Results

### CD8^+^ T cells transduced with high-avidity MDM2-TCR demonstrate reduced fitness in vitro

**FIGURE 1.**
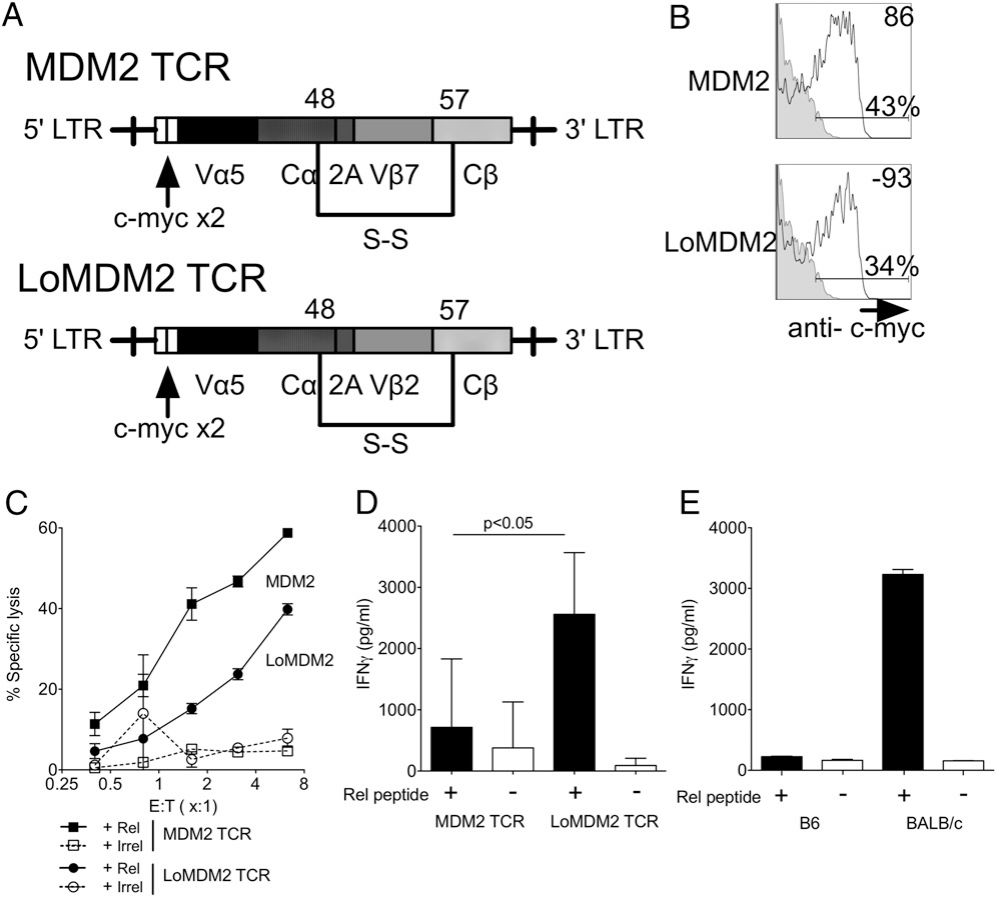
MDM2-TCR–transduced T cells demonstrate cytotoxicity but impaired cytokine generation. (**A**) Map of TCR gene sequences inserted into pMP71 retroviral vector. (**B**) Expression of c-myc tag used to track introduced TCR sequences in CD8^+^ T cells. Isotype controls are represented by gray-filled histograms. Data are representative of >20 independent experiments. (**C**) MDM2-TCR–transduced (squares) and LoMDM2-TCR transduced (circles) T cells were restimulated once before being incubated with ^51^Cr-labeled, peptide-loaded targets (filled symbols, relevant peptide; open symbols, control peptide) in a standard cytotoxicity assay. Data are representative of three independent experiments. Error bars represent SEM of triplicate values. (**D**) MDM2-TCR–transduced CD8^+^ T cells and LoMDM2-TCR–transduced CD8^+^ T cells were stimulated overnight with peptide-loaded targets on day 5 following transduction. IFN-γ concentrations within the culture supernatants were then evaluated by ELISA. Results are pooled from seven independent experiments. Error bars represent SD. (**E**) IFN-γ generation by BALB/c and B6 MDM2-TCR–transduced CD8^+^ T cells following overnight stimulation with peptide-loaded targets. Data are representative of two independent experiments. All statistically significant comparisons are depicted with the relevant *p* value; all other comparisons are not significantly different.

In initial experiments, we transduced purified CD8^+^ T cells from B6 splenocytes with retrovirus encoding codon-optimized TCRα and TCRβ genes derived from T clones recognizing an identical K^b^-restricted epitope of MDM2 (K^b^/pMDM100) with high (MDM2-TCR) or low avidity (LoMDM2-TCR). To avoid pre-existing tolerance, the K^b^-restricted CD8^+^ T cell clones were originally isolated from BALB/c splenocytes by in vitro stimulation with K^b^-positive stimulator cells coated with pMDM100 peptides (10). High-avidity T cell clones recognized lower peptide concentrations and killed MDM2-expressing tumors more effectively than did low-avidity clones (10). The TCR genes were isolated and modified to contain additional disulfide cysteine bonds between the constant α and β regions to improve TCR expression and to reduce mispairing with endogenous TCR chains (27) (Fig. 1A). c-myc tags were inserted at the N-terminal end of the TCR α-chains (Fig. 1A) (23). Use of c-myc–specific Abs allowed a comparison of TCR expression (Fig. 1B), whereas owing to predicted affinity differences, this comparison was not possible using K^b^/pMDM100 multimer reagents. Following transduction and in vitro expansion, T cells expressing the MDM2 or the LoMDM2-TCR both killed RMA-S target cells pulsed with relevant peptide (Fig. 1C). In contrast, the levels of naturally processed pMDM100 peptide in the tumor cell lines MBL-2 and C205 were sufficient to induce Ag-specific cytotoxicity by T cells expressing MDM2-TCR but not LoMDM2-TCR, consistent with the activity of the parental clones (11) (Supplemental Fig. 1). Although TCR-transduced CD8^+^ T cells recapitulated the cytotoxic function of the parental clones, this was not observed when effector cytokine production was tested (Fig. 1D). Whereas T cells expressing LoMDM2-TCR produced IFN-γ following stimulation overnight with RMA-S targets pulsed with relevant peptide, no cytokine production above background was observed when T cells expressing the MDM2-TCR were stimulated in the same way.

We hypothesized that the defective cytokine production of K^b^-positive B6 T cells was due to tolerance arising from cognate self-antigen recognition in vitro. Indeed, as an inhibitor of the cell cycle regulator p53, MDM2 protein expression in T cells is increased significantly during activation (28). To test this possibility, we transduced CD8^+^ T cells from B6 or BALB/c mice with the MDM2-TCR and tested their IFN-γ production in response to relevant peptide. BALB/c cells, lacking K^b^, are incapable of presenting pMDM100 to the MDM2-TCR. As shown in Fig. 1E, BALB/c but not B6 CD8^+^ T cells transduced with MDM2-TCR were able to produce IFN-γ after stimulation with relevant peptide.

**FIGURE 2.**
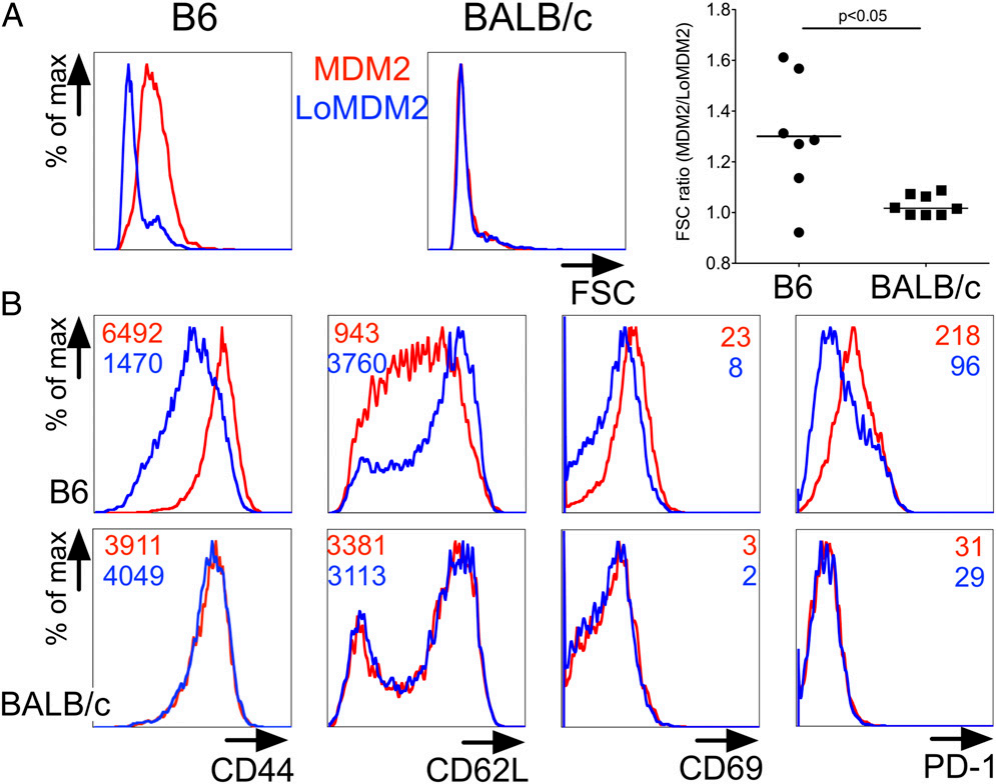
Ag recognition upon self by MDM2-TCR–transduced CD8^+^ T cells induces an activated PD-1^high^ phenotype. Splenocytes of B6 or BALB/c origin were transduced with the MDM2-TCR and LoMDM2-TCR. TCR-transduced T cells were then analyzed by flow cytometry. (**A**) Representative forward scatter (FSC) histogram of MDM2-TCR–transduced (red line) and LoMDM2-TCR–transduced (blue line) CD8^+^ T cells of B6 (*left plot*) and BALB/c (*right plot*) origin at 72 h after transduction. Graph shows ratio of median FSC of MDM2- to LoMDM2-transduced CD8^+^ T cells of B6 (●) or BALB/c (▪) origin. Each pair of datum points represents an independent experiment. (**B**) Flow cytometric histograms of surface expression of CD44, CD62L, CD69, and PD-1 of MDM2-TCR–transduced (red lines) or LoMDM2-TCR–transduced (blue lines) CD8^+^ T cells from B6 (*top row*) and BALB/c (*bottom row*) mice. Figures within each plot indicate mean fluorescence intensity (MFI) for each marker. Data are representative of two independent experiments. All statistically significant comparisons are depicted with the relevant *p* value; all other comparisons are not significantly different.

Supporting the notion that self-antigen recognition impaired function of MDM2-TCR–expressing CD8^+^ T cells, transduced cells had an activated phenotype even in the absence of target cells exogenously loaded with peptide. Thus, the forward scatter size of B6 CD8^+^ T cells expressing the MDM2-TCR was larger than that of B6 T cells expressing the LoMDM2-TCR or BALB/c T cells expressing the MDM2-TCR (Fig. 2A). Furthermore, within B6 T cells, expression of high-avidity MDM2-TCR was associated with elevated expression of the activation markers CD69, CD44, and PD-1 and with reduced expression of CD62L, whereas this was not the case in BALB/c T cells (Fig. 2B). Additional analysis of transduced B6 T cells showed that in the same cultures only the T cell subset expressing the MDM2-TCR displayed upregulation of PD-1, whereas the PD-1 expression level in gated MDM2-TCR^−^ B6 T cells was similar to that of BALB/c T cells (Supplemental Fig. 1). In addition to PD-1, B6 T cells expressing the MDM2-TCR expressed elevated levels of the inhibitory receptors LAG-3, 2B4, and NKG2A when compared with T cells expressing the F5-TCR (Supplemental Fig. 1). Finally, staining for annexin V and propidium iodide revealed an increased level of activation-induced apotosis when the MDM2-TCR was expressed in B6 T cells but not when it was expressed in BALB/c T cells (Supplemental Fig. 2).

Taken together, the in vitro data indicated that MDM2-TCR–transduced B6 T cells were able to display Ag-specific cytotoxicity. The data also indicated that, in the absence of exogenous peptide, stimulator cells capable of presenting endogenous MDM2 induced activation and apoptosis of freshly transduced T cells expressing the MDM2-TCR. This activated phenotype was associated with increased levels of LAG-3, 2B4, and NKG2A and impaired cytokine production when the T cells were further stimulated with peptide-pulsed stimulator cells.

### MDM2-TCR–transduced CD8 T cells lose cytotoxic functions upon transfer to Ag-expressing hosts

**FIGURE 3.**
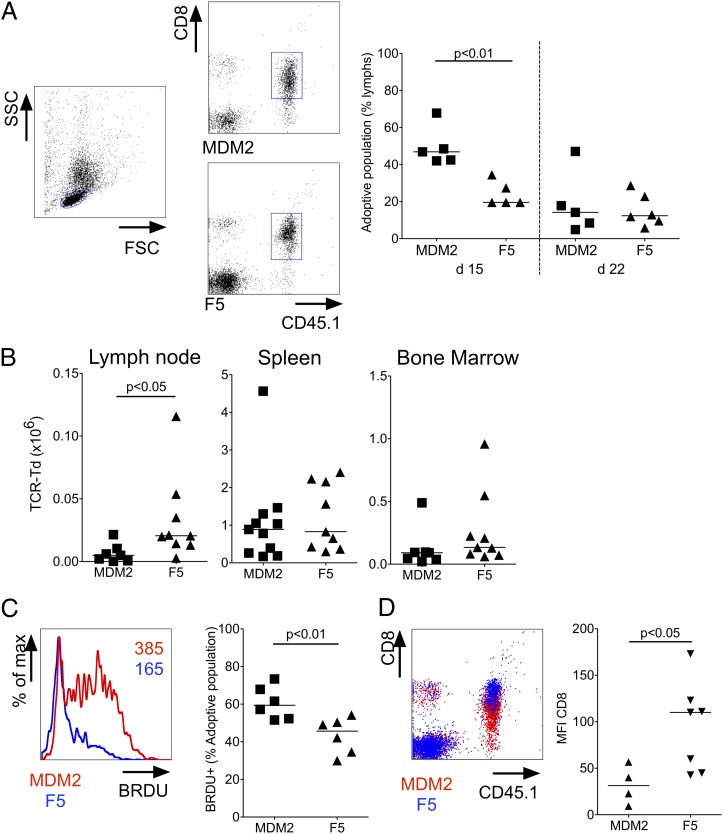
MDM2-TCR–transduced T cells tune CD8 expression upon transfer to Ag-expressing recipients. Immunomagnetically selected CD8^+^ T cells of B6 origin were transduced with the MDM2 or F5-TCR. Identical numbers of B6 MDM2-TCR–transduced or B6 F5-TCR–transduced CD8^+^ T cells were adoptively transferred to sublethally irradiated B6 mice. (**A**) A tail bleed was carried out on days 15 and 22 following transfer. The *left plot* shows the forward scatter (FSC) and side scatter (SSC) profile used to gate on lymphocytes. The *right plots* show representative CD45.1 and CD8 staining profiles used to identify the adoptively transferred lymphocytes in recipients of MDM2-TCR–transduced CD8^+^ T cells (*upper plot*) or of F5-TCR–transduced CD8^+^ T cells (*lower plot*). The graph on the *right* shows the frequency of adoptively transferred (CD45.1^+^CD8^+^) lymphocytes as a percentage of circulating lymphocytes at days 15 and 22 after transfer. (**B**) Absolute numbers of MDM2- and F5-TCR–transduced CD8 T cells populations in lymph nodes, spleen, and bone marrow 4 wk after transfer. Data are pooled from four independent experiments. (**C**) Proliferation of MDM2-transduced (red) and F5-TCR–transduced (blue) CD8 T cell populations within the spleen was determined by BrdU incorporation after oral administration of BrdU between days 24 and 29 after transfer. Representative flow cytometric histograms of BrdU staining (*left*, numbers represent mean fluorescence intensity [MFI] of BrdU within adoptive population) and summary data (*right*) are shown. (**D**) Surface expression of CD8β on circulating MDM2-TCR–transduced (CD45.1^+^c-myc^+^) and F5-TCR–transduced cells (CD45.1^+^ Vβ11^+^) at day 22 following transfer. Representative flow cytometric histograms of CD8β versus CD45.1 staining (*left*) and summary data of MFI of CD8β on MDM2-TCR–transduced (▴) and F5-TCR–transduced (▼) cells are shown (*right*). All statistically significant comparisons are depicted with the relevant *p* value; all other comparisons are not significantly different.

To determine the in vivo fate of CD8^+^ T cells expressing the MDM2-TCR, we adoptively transferred B6 T cells (CD45.1) to sublethally irradiated B6 CD45.2 hosts. As a control, we used CD8^+^ T cells expressing the F5-TCR (Vβ11^+^) specific for an influenza-derived epitope, as these were not affected by recognition of self-antigen. Peripheral blood taken at day 15 following transfer showed that the relative frequency of MDM2-TCR–expressing CD8^+^ T cells was higher than F5-TCR–expressing T cells, although by day 22, the percentage was similar in the two groups of mice (Fig. 3A). At day 29, the absolute numbers of MDM2- and F5-TCR–transduced T cells were similar in spleen and bone marrow, whereas lymph nodes showed a greater number of F5-TCR–transduced T cells (Fig. 3B). To evaluate in vivo turnover of TCR-transduced CD8^+^ populations, mice received BrdU in their drinking water between days 24 and 29. As shown in Fig. 3C, CD8^+^ T cells expressing the MDM2-TCR showed greater BrdU incorporation compared with T cells expressing the F5-TCR, indicative of greater turnover of the former population. Although CD8 expression was equivalent at transfer, we consistently observed reduced expression in T cells expressing the MDM2-TCR compared with control T cells at days 15 (data not shown) and 22 (Fig. 3D), a finding consistent with tuning of coreceptor expression in the presence of Ag (29, 30).

**FIGURE 4.**
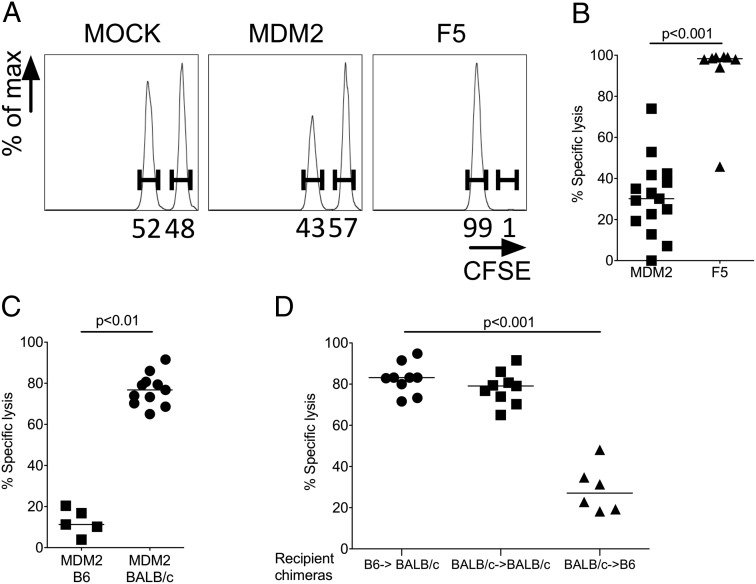
MDM2-TCR–transduced T cells demonstrate reduced cytotoxicity after transfer to Ag-expressing recipients. The Ag-specific function of adoptively transferred MDM2- and F5-TCR–transduced CD8^+^ T cells in B6 hosts was assessed on days 26–29 after transfer by means of an in vivo cytotoxicity assay. This compared lysis of pMDM100-loaded (CFSE^low^) against pNP366-loaded (CFSE^high^) B6 B cell targets in the spleen 16 h after the B cells were injected i.v. as a 1:1 mix. (**A**) Representative histograms showing each target population within spleens of recipients of mock, MDM2-TCR– and F5-TCR–transduced CD8^+^ T cells. A change in the ratio of these populations relative to one another indicates specific lysis of one target population. This is calculated as percentage specific lysis, as described in *Materials and Methods*. (**B**) Summary specific lysis of B cell targets in recipients of MDM2-TCR– and F5-TCR–transduced CD8^+^ T cells. Data are from five independent experiments. (**C**) B6 or BALB/c MDM2-TCR–transduced CD8^+^ T cells (0.75–1 × 10^6^) were transferred to sublethally irradiated B6 or BALB/c hosts, respectively, and on days 26–29 in vivo cytotoxicity was determined as before, although using CB6F1 target cells pulsed with pMDM100 or pNP366 peptide. Summary data from three independent experiments are shown. (**D**) BALB/c-derived (Thy1.1) T cells that were tolerant to K^b^ (see *Materials and Methods*) were transduced with the MDM2-TCR. Of these cells, 0.2–0.5 × 10^6^ were transferred into sublethally irradiated recipient chimeras in which cognate Ag was either restricted to the hematopoietic compartment (B6→BALB/c), completely absent (BALB/c→BALB/c), or restricted to the nonhematopoietic compartment (BALB/c→B6). Fifteen days after transfer, an in vivo cytotoxicity assay was performed using CB6F1 B cells pulsed with pMDM100 or pNP366 peptide and summary data are shown (pooled from three independent experiments). All statistically significant comparisons are depicted with the relevant *p* value; all other comparisons are not significantly different.

Although these data were indicative that the MDM2-TCR but not the F5-TCR was triggered by Ag in vivo, analysis of peripheral tissues demonstrated absence of immunopathology (Supplemental Fig. 4) and, importantly, as a global measure of health, there were no differences in recipient weights between the groups. This suggested that MDM2-TCR–transduced CD8^+^ T cells did not effect tissue damage upon Ag encounter in vivo. Indeed, when we tested the capacity for each TCR-transduced population to induce in vivo cytotoxicity against B cells pulsed with relevant peptide, we noted nearly complete lysis of pNP366-loaded cells in recipients of F5-TCR T cells, but only partial lysis of the pMDM2-loaded targets in recipients of MDM2-TCR T cells (Fig. 4A, 4B). To confirm that the dysfunction of MDM2-TCR–transduced CD8^+^ T cells was a consequence of self-antigen exposure, we compared in vivo cytotoxicity of MDM2-TCR–transduced B6 or BALB/c T cells following their transfer to B6 or BALB/c hosts, respectively. As shown in Fig. 4C, specific cytotoxicity was significantly greater in Ag-free BALB/c mice compared with Ag-expressing B6 hosts. Therefore, upon transfer to Ag-expressing hosts, CD8^+^ T cells expressing the MDM2-TCR are not deleted but rather tune down CD8 coreceptor expression and lose cytotoxic function.

We next explored whether in vitro Ag exposure was required for the observed reduction of effector function in vivo. Lethally irradiated CB6F1 mice were reconstituted with a mixture of *Rag^−/−^* B6 bone marrow cells and T cell–depleted BALB/c bone marrow cells (see *Materials and Methods*). The T cells of the reconstituted F_1_ mice were of BALB/c origin, tolerant to the H2^b^ haplotype, and unable to present the H2-K^b^–restricted pMDM100. These T cells were isolated, transduced with the MDM2-TCR in vitro, and adoptively transferred into chimeric recipients presenting the TCR-recognized Ag in hematopoietic or nonhematopoietic tissues. As expected, the transferred T cells showed strong cytotoxicity in BALB/c→BALB/c recipients where there was an absence of TCR-recognized Ag (Fig. 4D). Cytotoxic function was retained in mice presenting pMDM100 in the hematopoietic compartment (Fig. 4D, B6→BALB/c recipient chimeras), but was substantially reduced when Ag presentation occurred in nonhematopoietic tissues (BALB/c→B6 recipient chimeras). These experiments indicate nonhematopoietic Ag induced T cell dysfunction, and that this dysfunction did not depend on Ag presentation during T cell transduction in vitro.

### Provision of CD4^+^ T cell help enhances cytotoxic function of MDM2-TCR–transduced CD8^+^ T cells

**FIGURE 5.**
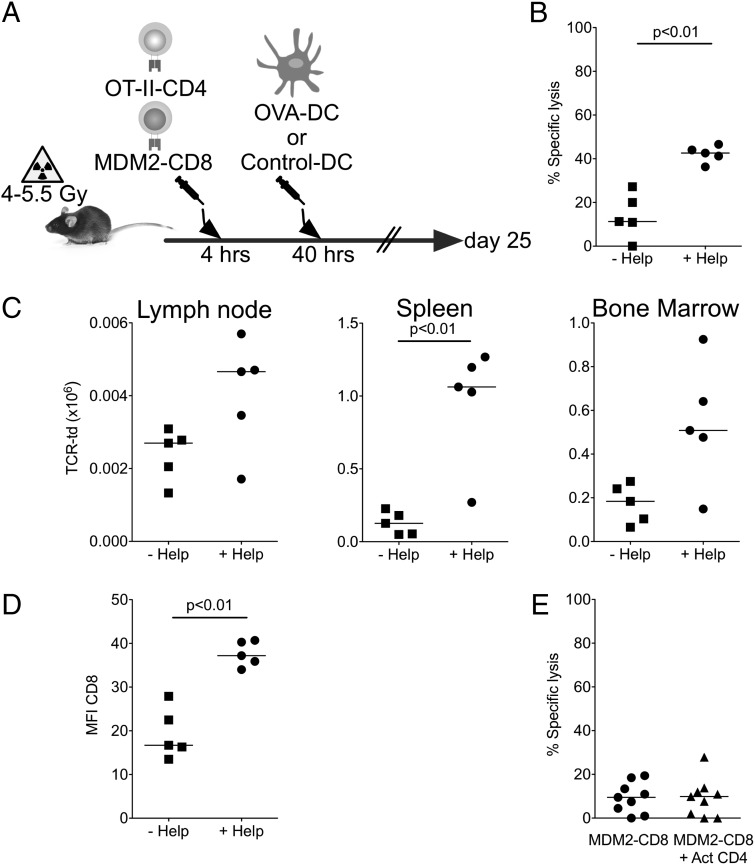
Provision of heterologous help improves the functions of MDM2-TCR–transduced CD8^+^ T cells in vivo. (**A**) MDM2-TCR–transduced CD8^+^ T cells (0.5 × 10^6^) and 0.5 × 10^6^ OT-II CD4^+^ T cells were cotransferred to sublethally irradiated B6 mice. Forty hours later, recipient mice received 0.5 × 10^6^ LPS-matured DCs loaded with pOVA323 or control peptide. (**B**) On day 25 following transfer, Ag-specific responses of MDM2-TCR–transduced CD8^+^ T cells were assessed by an in vivo cytotoxicity assay against peptide-loaded B cell targets determined as previously described. (**C**) Absolute number of adoptively transferred MDM2-TCR–transduced CD8^+^ T cells in secondary lymphoid organs at day 25. (**D**) Summary data of surface expression of CD8β on circulating MDM2-TCR–transduced (CD45.1^+^c-myc^+^) T cells within the spleen were determined on day 25. Data in (A)–(D) are pooled from two independent experiments. (**E**) MDM2-TCR–transduced CD8^+^ T cells and CD4^+^ T cells (which had been activated with CD3/CD28 beads for 24 h) were transferred to sublethally irradiated B6 mice and in vivo cytotoxicity against peptide-loaded B cell targets was determined as before at 28 d following transfer (data pooled from two independent experiments). All statistically significant comparisons are depicted with the relevant *p* value; all other comparisons are not significantly different.

To determine whether MDM2-TCR–transduced CD8^+^ T cell cytotoxicity could be restored through provision of help, we transferred MDM2-TCR–transduced CD8^+^ T cells to conditioned B6 recipients together with OVA-specific *Rag^−/−^* OT-II–TCR transgenic CD4^+^ T cells (Fig. 5A). Following T cell transfer, mice were immunized with B6 DCs loaded with pOVA323 peptides or irrelevant peptides. Recipient mice receiving DCs pulsed with pOVA323 peptide demonstrated a significant increase in in vivo cytotoxicity compared with mice receiving DCs loaded with control peptide (Fig. 5B). The observed increase in cytotoxicity was associated with an increase in MDM2-TCR–transduced CD8^+^ T cell numbers in the spleen, with a similar trend in the bone marrow and lymph nodes (Fig. 5C), and an increase in CD8 coreceptor expression within the MDM2-TCR^+^ T cell population (Fig. 5D). To further demonstrate that cognate recognition by CD4^+^ T cells was required for the provision of help in vivo, we adoptively transferred polyclonal mock-transduced CD4^+^ T cells. As shown in Fig. 5E, polyclonal CD4^+^ T cells did not improve cytotoxicity of MDM2-TCR–transduced CD8^+^ T cells.

### CD4^+^ T cells transduced with the MDM2-TCR partially overcome tolerance of CD8^+^ T cells transduced with same TCR

**FIGURE 6.**
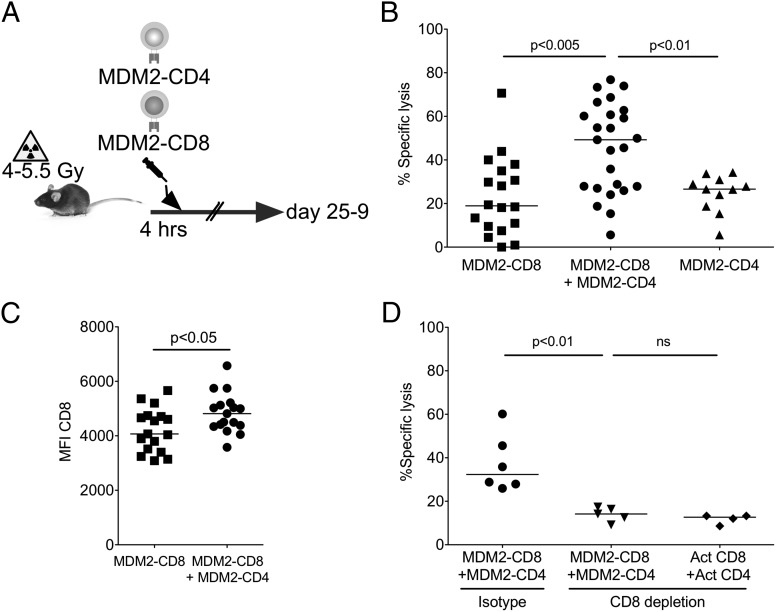
Transfer of MDM2-TCR–transduced CD4^+^ T cells improves the functions of MDM2-TCR–transduced CD8^+^ T cells in vivo. (**A**) MDM2-TCR–transduced CD8^+^ T cells (0.5–1 × 10^6^) with or without the same number of MDM2-TCR–transduced CD4^+^ T cells were transferred to sublethally irradiated B6 mice. A further group received 0.5–1 × 10^6^ MDM2-TCR–transduced CD4^+^ T cells without CD8^+^ T cells. (**B**) Ag-specific responses of the adoptive populations were determined using an in vivo cytotoxicity assay against peptide-loaded B cell targets at 25–29 d following transfer. Data are pooled from five independent experiments. (**C**) Summary data of surface expression of CD8β on splenic MDM2-TCR–transduced CD8^+^ T cells at days 25–29 after transfer without or with MDM2-TCR–transduced CD4^+^ T cells. Data are pooled from five independent experiments. (**D**) Conditioned B6 recipients received 0.5 × 10^6^ MDM2-TCR–transduced CD8^+^ T cells and either 0.5 × 10^6^ MDM2-TCR–transduced CD4^+^ or 0.5 × 10^6^ CD3/CD28 bead–activated polyclonal CD4^+^ T cells. The indicated cohorts were also treated with weekly i.p. injections of a CD8-depleting or isotype Ab. CD8 depletion was >95%. An in vivo cytotoxicity assay was carried out on day 25, and the specific lysis of pMDM2-loaded targets was determined as before. Data are pooled from two independent experiments. All statistically significant comparisons are depicted with the relevant *p* value; all other comparisons are not significantly different.

We have shown previously that CD4^+^ T cells bearing an MHC-I–restricted TCR (F5) can enhance the antitumor activity of CD8^+^ T cells bearing the same TCR using a model Ag that is tumor-specific and not expressed in normal tissues (17). We were therefore interested whether CD4^+^ T cells engineered to express the MDM2-TCR could rescue the function of CD8^+^ T cells rendered hyporesponsive by a naturally expressed TAA in normal tissues. Using the experimental design in Fig. 6A, we transferred MDM2-TCR–transduced CD8^+^ T cells or MDM2-TCR–transduced CD4^+^ T cells or both populations together to conditioned B6 mice and performed in vivo cytotoxicity assays as before. As shown in Fig. 6B, we observed significantly greater cytotoxicity against pMDM100-pulsed targets where both transduced CD4^+^ and CD8^+^ T cells were transferred. As in the OVA help model, we consistently observed an increase in CD8 coreceptor expression within the transduced CD8^+^ T cell population upon cotransfer of CD4^+^ and CD8^+^ T cells compared with mice that received CD8^+^ T cells in isolation (Fig. 6C).

From these data, however, it was not possible to distinguish whether MDM2-TCR–transduced CD4^+^ T cells were providing help or contributed directly to the killing of pMDM100-loaded targets. To investigate this, we transferred MDM2-TCR–transduced CD8^+^ T cells and an equal number of either MDM2-TCR–transduced or mock-transduced CD4^+^ T cells into Ag-expressing hosts. Additionally, we also administered a CD8-depleting monoclonal or isotype control Ab throughout the experiment. In mice receiving CD8-depleting Ab, any residual cytotoxic activity could be attributed to the transferred CD4^+^ T cell population alone or other non-CD8 populations in the host. In the presence of CD8 depletion, in vivo cytotoxicity of pMDM100-pulsed target cells was not different in recipients of MDM2-TCR–transduced CD4^+^ T cells or mock-transduced CD4^+^ T cells (Fig. 6D), indicating that the MDM2-TCR–expressing CD4^+^ T cells did not display specific killing above the background seen with polyclonal CD4^+^ T cells.

Whereas the Ag-specific cytotoxicity of MDM2-TCR–transduced CD8^+^ T cells was improved in the presence of MDM2-TCR–transduced CD4^+^ T cells, there was no evidence of immunopathology in mice receiving both of these populations. Specifically, there was weight loss in recipients of MDM2-TCR–transduced CD4^+^ and CD8^+^ T cell populations compared with mice receiving F5-TCR–transduced CD4^+^ and CD8^+^ T cells, and, additionally, the histologies of liver, lung, skin, and colon from these recipient mice were normal (Supplemental Fig. 3).

### Cointroduction of the CD8 coreceptor blocks the helper functions of MDM2-TCR–expressing CD4^+^ T cells

**FIGURE 7.**
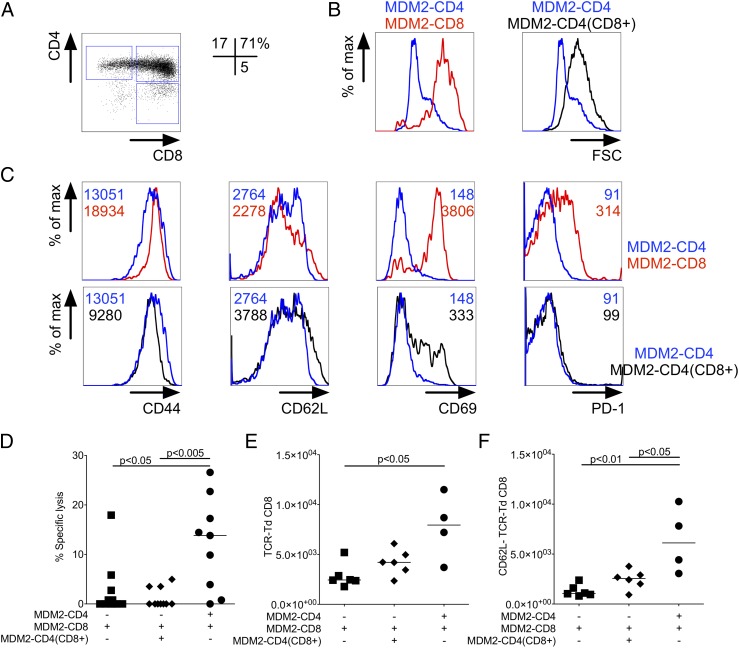
MDM2-TCR–transduced CD4^+^ T cells coexpressing CD8αβ coreceptor cannot provide help to tolerant MDM2-TCR–transduced CD8^+^ T cells. (**A**) CD4 T cells were cotransduced with MDM2-TCR and CD8αβ. Representative CD4 and CD8β staining of gated c-myc^+^ (MDM2-TCR^+^) cells is shown. Percentages refer to the populations within the depicted gates. (**B**) Representative histograms of forward scatter (FSC) at day 3 following transduction of MDM2-TCR–transduced CD4^+^ T cells (blue line) compared with MDM2-TCR–transduced CD8^+^ T cells (red line) or MDM2-TCR–transduced CD4^+^ T cells cotransduced with CD8αβ (black line). (**C**) Representative staining profiles of CD44, CD62L, CD69, and PD-1 expression in gated c-myc^+^ (MDM2-TCR^+^) CD4^+^ T cells (blue line), CD8^+^ T cells (red line), or CD4^+^ T cells cotransduced with CD8αβ (black line). (**D**) MDM2-TCR–transduced CD8^+^ T cells (1 × 10^6^) were transferred to sublethally irradiated B6 hosts with or without the same number of CD4^+^ T cells cotransduced with MDM2-TCR and CD8αβ or transduced with the MDM2-TCR alone. At day 25 after transfer an in vivo cytotoxicity assay was carried out against peptide-loaded B cell targets (data pooled from two independent experiments). (**E**) Absolute numbers and (**F**) CD62L staining profile of splenic MDM2-TCR–expressing CD8^+^ T cells in each of the indicated experimental groups. All statistically significant comparisons are depicted with the relevant *p* value; all other comparisons are not significantly different.

Unlike MDM2-TCR–transduced CD8^+^ T cells, CD4^+^ T cells expressing the same TCR did not demonstrate increased activation as determined by increased cell size (Fig. 7B). If lack of the CD8 coreceptor in MDM2-TCR–transduced CD4^+^ T cells permitted them to avoid tolerance and confer help, then introduction of CD8α and CD8β coreceptor chains may impede their ability to help (Fig. 7A). Consistent with recognition of MDM2, the cotransduction of CD8αβ and MDM-TCR into B6 CD4^+^ T cells resulted in increased cell size, although less than the size seen in B6 CD8^+^ T cells transduced with the MDM2-TCR only (Fig. 7B). Similarly, upregulation of the CD69 activation marker was less pronounced in CD4^+^ T cells expressing TCR plus CD8 compared with CD8^+^ T cells expressing the TCR only (Fig. 7C). Unlike CD8^+^ T cells, the TCR^+^ CD8–expressing CD4^+^ T cells did not show upregulation of activation markers CD44 and PD-1. This in vitro analysis showed that the self-antigen–triggered T cell activation was more effective in CD8^+^ T cells compared with CD4^+^ T cells expressing the same TCR plus the CD8 coreceptor.

Despite apparently reduced activation in vitro, the in vivo analysis revealed that TCR plus CD8 transfer abolished the ability of the CD4^+^ T cells to provide helper function. Whereas CD4^+^ T cells expressing TCR only improved the cytotoxicity of CD8^+^ T cells, this was not the case with transfer of CD4^+^ T cells expressing TCR plus CD8 (Fig. 7D). Further analysis showed that help provided by TCR-transduced CD4^+^ T cells increased the number of MDM2-TCR–transduced CD8^+^ T cells (Fig. 7E); this increase seemed primarily due to an accumulation of activated CD8^+^ T cells that lost expression of CD62L (Fig. 7F). The increase in CD8^+^ T cell numbers and loss of CD62L expression were not seen when CD4^+^ T cells were transduced with the MDM2-TCR plus CD8. Taken together, the data show that the dysfunction of MDM2-TCR–transduced CD8^+^ T cells in Ag-expressing hosts can be partially reversed by cotransfer of CD4^+^ T cells engineered to express an identical TCR, where the absence of the CD8 coreceptor enables preservation of Th function.

## Discussion

To our knowledge, we have shown for the first time that an identical MHC-I–restricted TCR can confer tolerance or helper function upon transfer to CD8^+^ and CD4^+^ T cells, respectively. Absence of the CD8 coreceptor was critical in enabling CD4^+^ T cells to maintain helper functions and avoid dysfunction upon interaction with MHC-I–presented peptide ligands.

Our data indicate the in vivo induction of unresponsiveness mediated by a TCR specific for a tumor-associated self-antigen was dependent on the presence of CD8. CD8 improves TCR signaling by increasing the stability of the TCR binding to MHC/peptide and enhancing the availability of p56^Lck^ at the immunological synapse (31). Thus, the data presented in the present study indicate that improved T cell stimulation resulted in tolerance induction, whereas weak T cell stimulation maintained immune function despite chronic Ag exposure. This is supported by in vitro data showing that both B6 origin CD8^+^ T cells expressing the MDM2-TCR and CD4^+^ T cells expressing TCR plus CD8 were activated by self-antigen, whereas this was not the case when CD4^+^ T cells expressed TCR alone. The lack of robust in vitro activation of TCR-transduced CD4^+^ T cells may be due to insufficient sensitivity or relevance of the assays used, whereas chronic in vivo exposure to MDM2 Ag may sustain weak TCR signals that are sufficient for helper functions. Other weak near threshold TCR signals in CD4^+^ T cells leading to in vivo functional outputs in CD4^+^ T cells include clonal competition in the periphery in response to lymphopenia (32). The notion that reducing TCR signal strength may improve in vivo function is also supported by a study showing that artificial lowering of p56^Lck^ availability in CD4^+^ T cells improved their capacity to generate helper cytokines in response to chronic stimulation by a TAA (33).

Upon in vivo transfer to Ag-expressing hosts, MDM2-TCR–transduced CD8^+^ T cells were not deleted. Instead, they demonstrated increased turnover and CD8 downtuning consistent with ongoing antigenic stimulation (34, 35). Tuning of CD8 expression is also observed in other models of peripheral tolerance, where TCR stimulation interrupts the normal homeostatic regulation that maintains CD8 transcription via IL-7R– and STAT5-mediated signaling (35). CD8 tuning may explain the lack of autoimmune pathology in this model despite transfer of high-avidity T cells reactive to a widely expressed self-antigen.

Although removal from Ag and exposure to common γ cytokines may reset CD8 coreceptor expression and increase functional TCR avidity (35), these strategies are unlikely to be applicable in a clinical context. We therefore evaluated cotransfer of conventional MHC-II–restricted CD4^+^ T cells or MHC-I–restricted T cells expressing the MDM2-TCR. In both cases, help provided by CD4^+^ T cells increased CD8 coreceptor expression in MDM2-specific CD8^+^ T cells, which was associated with increased in vivo cytotoxicity against peptide-pulsed target cells, in the absence of detectable reactivity against normal tissues with low expression of MDM2.

Tolerance induction in MDM2-TCR–expressing CD8^+^ T cells was driven by interactions with the nonhematopoietic compartment. Indeed, when MDM2 Ag presentation was limited to hematopoietic cells, their immune function was preserved. Although there are many possible mechanisms contributing to the tolerogenic function of nonhematopoietic tissues, we would like to speculate on a role of costimulation. For example, costimulatory ligands such as CD80, CD86 and CD154 are more abundant on hematopoietic host cells, whereas nonhematopoietic tissues may provide cognate TCR stimulation without providing costimulation. The presence of signal 1 without signal 2, such as in the latter scenario, has previously been linked to tolerance induction (36). In CD4^+^ T cells expressing only the MDM2-TCR, a weak TCR signal 1 combined with a strong signal 2 delivered by professional APCs appeared sufficient to maintain helper function without induction of tolerance. Furthermore, enhancing TCR signaling through forced expression of CD8 appeared sufficient to lead to tolerance induction in such CD4^+^ T cells.

MDM2 is a suitable target TAA, as it is involved in oncogenesis and is overexpressed in a high proportion of cancers (6–8). However, MDM2 is also present in normal tissues and its expression sharply increases following T cell activation (37), when it acts via p73 to inhibit transcription of Bim and prevent activation-induced cell death (28). This could increase endogenous presentation of K^b^/pMDM100 by T cells, leading to recognition through the transduced TCR and T cell killing. Indeed, this was observed following transduction of T cells with other allorestricted TCRs. Thus, HLA-A2^+^ CD8 T cells transduced with HLA-A2–restricted TCRs specific for two TAAs, survivin and HMMR, demonstrated fratricide in vitro as a result of increased protein expression following T cell activation (38). In our experiments, we were unable to demonstrate fratricide of MDM2-TCR–transduced T cells in vitro or in vivo.

In conclusion, our data indicate a potential means for overcoming tolerance of CD8^+^ T cells to a TAA. Upon transfer of CD8^+^ T cells to Ag-expressing hosts, induction of dysfunction by TAA exposure on normal tissues may be overcome by transfer of CD4^+^ T cells bearing the same TCR.

## Supplementary Material

Data Supplement
