# Supplementary material for: CD8 T Cell Tolerance to a Tumor-Associated Self-Antigen Is Reversed by CD4 T Cells Engineered To Express the Same T Cell Receptor
Source: J Immunol. 2014 Dec 24;194(3):1080–9. doi: 10.4049/jimmunol.1401703 (PMC4298128; doi:10.4049/jimmunol.1401703)
Supplement: Data Supplement [file JI_1401703.zip › JI_1401703_Supplemental_Figures_1.pdf]

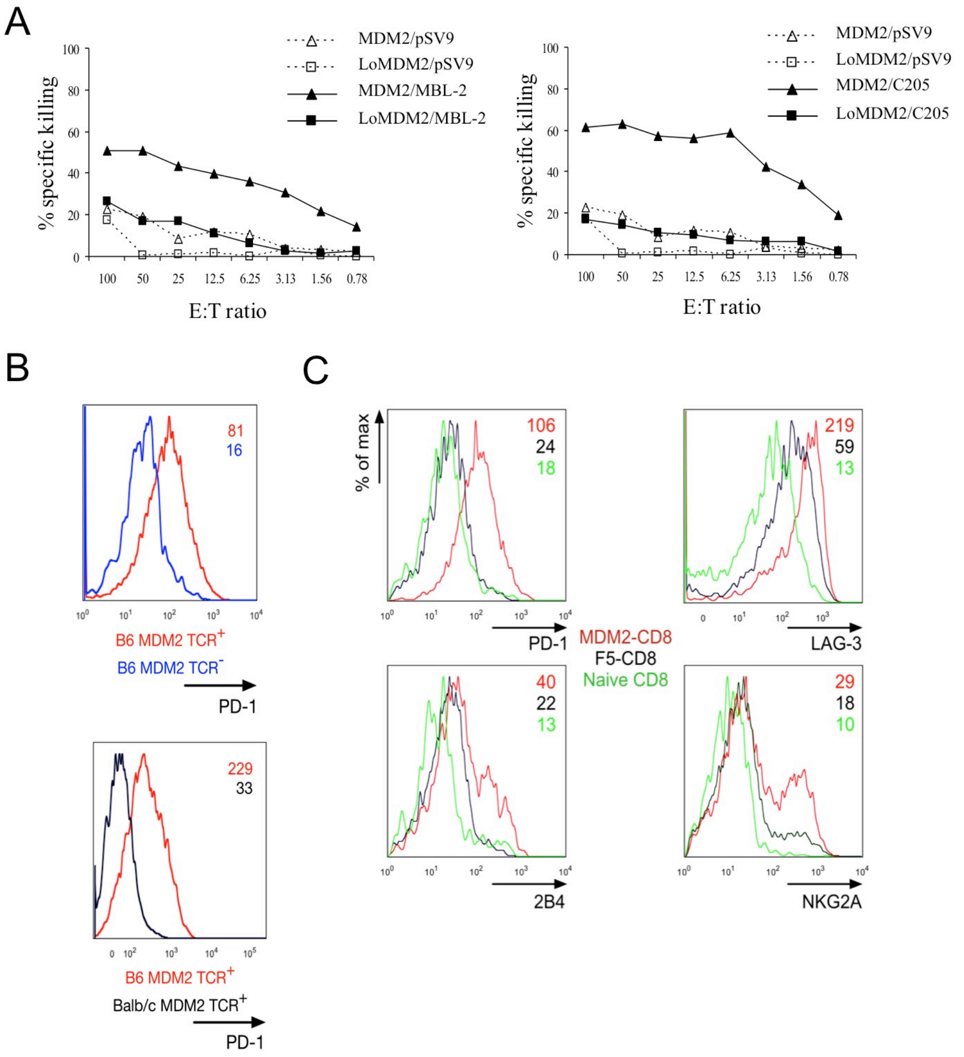

Supplementary Figure 1. Recognition of endogenous peptide by MDM2-TCR expressing T cells. (A) MDM2-TCR and LoMDM2-TCR transduced T cells were tested for their ability to kill $H2^b$ MDM2-expressing tumor cell lines MBL-2 (*left*) and C205 (*right*). MBL-2 and C205 tumor cells were used because they were also killed by the original CTL from which the MDM2-TCR was isolated (see ref. 11 for analysis of the original CTL). RMA-S cells coated with the irrelevant peptide (pSV9) were included as a control for non-specific background killing. (B) Flow cytometric analysis of PD-1 expression was carried out 7 days post transduction of splenocytes from C57BL/6 (B6) or BALB/c mice. *Top-* Representative histograms show the PD-1 staining profile of B6 splenocytes after gating on $CD8^+$/c-myc^+ (MDM2-TCR^+) T cells (red) or $CD8^+$/c-myc^- (MDM2-TCR^-) T cells (blue). *Bottom-* PD-1 staining profile of gated $CD8^+$/c-myc^+ B6 T cells (red) compared to gated $CD8^+$/c-myc^+ BALB/c T cells (black). (C) Flow cytometric analysis of expression of PD-1, LAG-3, 2B4, NKG2A was carried out 7 days post transduction of B6 splenocytes. Cells were transduced with MDM2-TCR (red line), F5-TCR (black line) and compared to naïve un-transduced T cells (green line). Representative histograms are shown of the expression of the indicated molecules on gated live $CD8^+$/c-myc^+ (MDM2-TCR transduced) or $CD8^+$/$V\beta11^+$ (F5 TCR transduced) T cells. Numbers in the top right of each histogram are the median fluorescence intensity (MFI) of that marker on the gated T cell population. Results are representative of at least 2 independent experiments

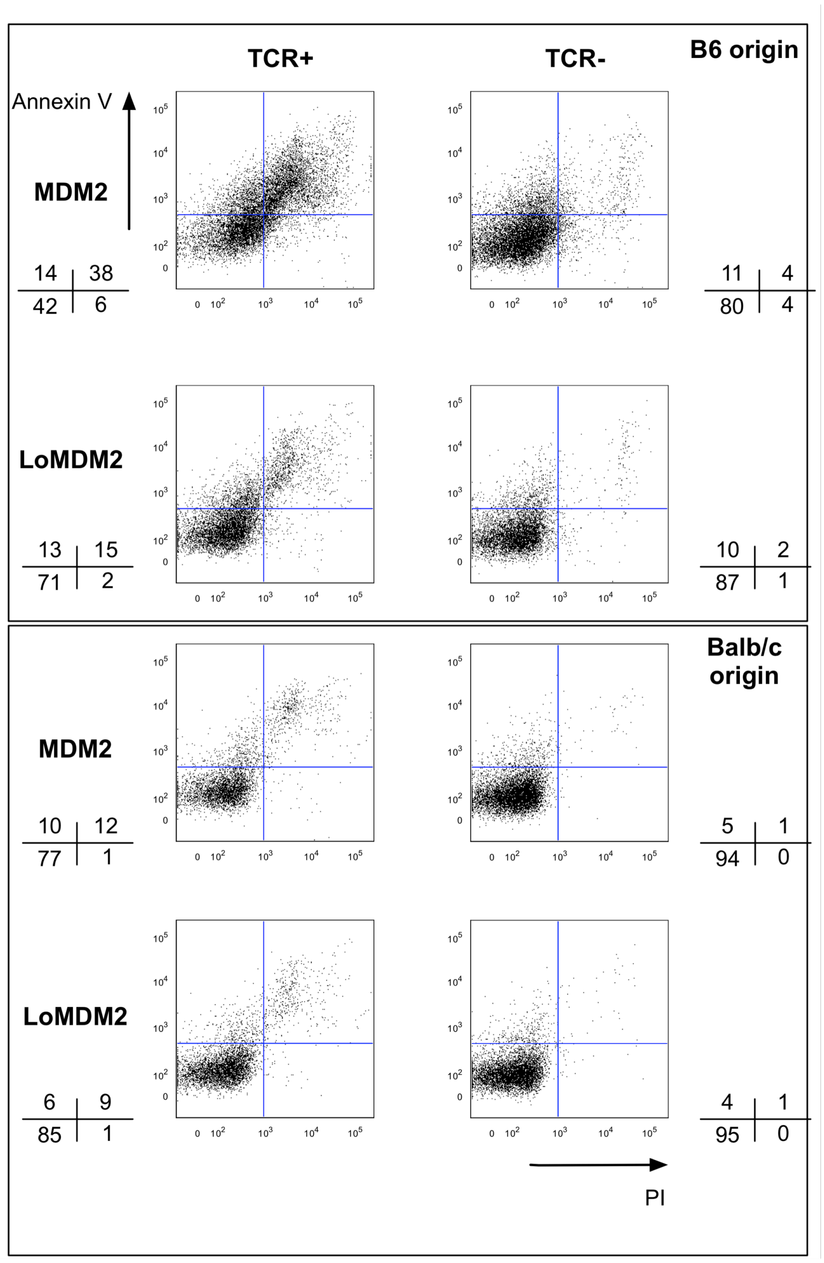

Supplementary Figure 2. Enhanced apoptosis in MDM2 TCR transduced CD8+ T cells of B6 mice. B6 and BALB/c splenocytes were transduced with MDM2 and LoMDM2-TCRs. On day 4 post transduction gated CD8+ T cells were analyzed for annexin V and propidium iodide (PI) staining. Plots show the proportion of T cells in early (upper left) and late (upper right) apoptosis, gated in the left hand plots on c-myc+ (TCR+) T cells, c-myc- (TCR-) cells in the right-hand plots. Data are representative of 2 independent experiments.

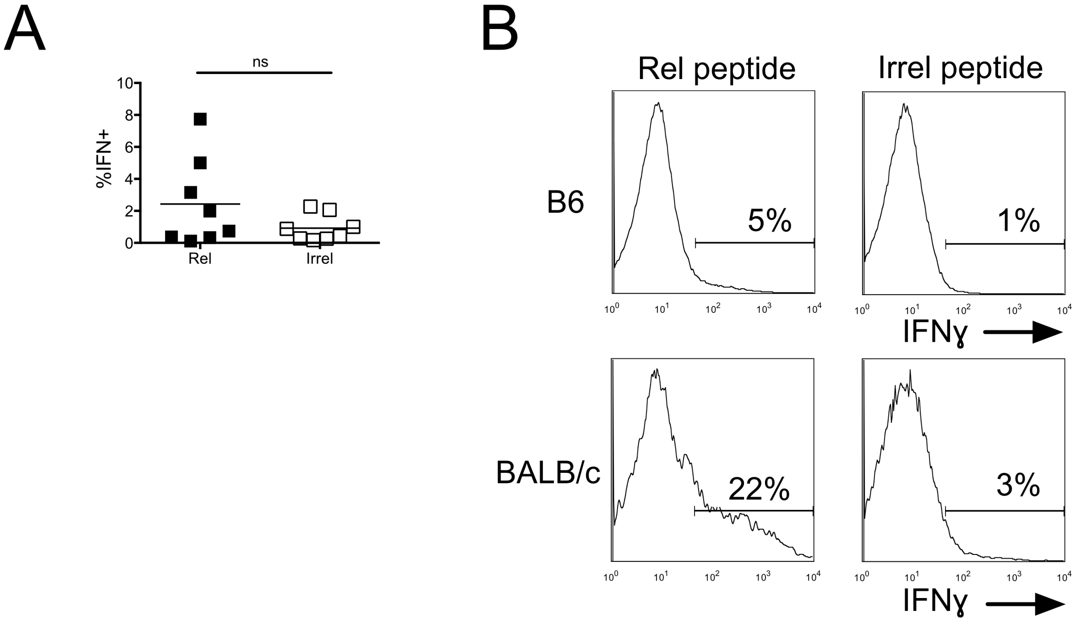

Supplementary Figure 3. MDM2-TCR-transduced CD8$^+$ T cells from BALB/c but not B6 mice show antigen-specific IFNγ production after stimulation with pMDM100-loaded targets. B6 or BALB/c (H2-K^b negative) splenocytes were transduced with MDM2-TCR. On day 5 post transduction, they were stimulated with RMA-S targets loaded with relevant pMDM100 peptide (Rel peptide) or control peptide (Irrel peptide) overnight and the IFNγ response determined by intracellular cytokine staining. (A) Summary results from 5 experiments of the IFNγ response from B6-derived MDM2-TCR transduced CD8+ T cells. (B) Representative histograms of intracellular IFNγ staining of MDM2-TCR transduced B6 and BALB/c CD8+ T cells are shown. These results are representative of 2 independent experiments.

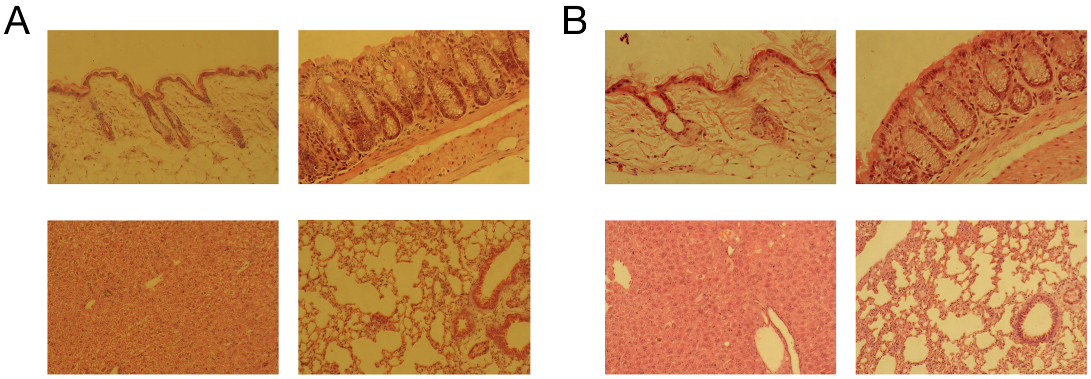

Supplementary Figure 4. Recipients of MDM2-TCR transduced T cells do not show histological evidence of immunopathology MDM2-TCR transduced $CD8^+$ T cells with or without the same number of MDM2-TCR transduced $CD4^+$ T cells were transferred to sub-lethally irradiated B6 mice. At 25-29 days following transfer, the mice were sacrificed and samples of skin, liver, lung and colon were taken for histopathological assessment, which was carried out in a single blind fashion. There was no detectable tissue pathology in mice treated with MDM2-TCR $CD8^+$ T cells, or MDM2-TCR $CD8^+$ and $CD4^+$ T cells. (A) Representative histology sections of skin (upper left), colon (upper right), liver (lower left), lung (lower right) of recipients of MDM2-TCR transduced $CD8^+$ T cells. (B) Representative histology sections of skin (upper left), colon (upper right), liver (lower left), and lung (lower right) of recipients of MDM2-TCR transduced $CD4^+$ and $CD8^+$ T cells.
